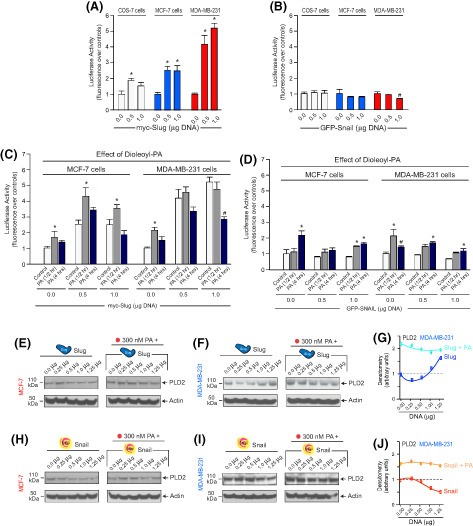# Corrigendum to: The transcription factors slug (SNAI2) and snail (SNAI1) regulate phospholipase D (PLD) promoter in opposite ways towards cancer cell invasion

**DOI:** 10.1002/1878-0261.13402

**Published:** 2023-03-18

Ramya Ganesan et al. [1] would like to correct Fig. 5F as errors were introduced in the preparation of this figure for publication. The authors were able to provide to the journal the raw data and the corrected figure panel. The EGFR blot shown in Fig. 3 panel (G) was inadvertently duplicated and misplaced as the actin blot in Fig. 5 panel (F). The journal has reviewed the original raw data and verified the error. The Editors and Authors confirm that the correction of this error has not altered the interpretation of data and did not affect the conclusions presented in the article. The authors sincerely apologise for their mistake and for any inconvenience caused.

The corrected figure is reproduced below.